# Beyond Processing: Furin as a Central Hub in Viral Pathogenesis and Genetic Susceptibility

**DOI:** 10.3390/biom15111530

**Published:** 2025-10-30

**Authors:** Adrián Alejandro Silva-Ríos, Carlos Ernesto Mora-Ornelas, Luna Galilea Flores-Medina, José Francisco Muñoz-Valle, Carlos Daniel Díaz-Palomera, Mariel García-Chagollan, Alexis Missael Vizcaíno-Quirarte, Oliver Viera-Segura

**Affiliations:** 1 Licenciatura en Médico Cirujano y Partero, Centro Universitario de Ciencias de la Salud, Universidad de Guadalajara, Sierra Mojada No. 950, Col. Independencia, Guadalajara 44340, Mexico; adrian.silva9121@alumnos.udg.mx (A.A.S.-R.); lunafmd04@gmail.com (L.G.F.-M.); 2 Doctorado en Ciencias Biomédicas, Departamento de Fisiología, Centro Universitario de Ciencias de la Salud, Universidad de Guadalajara, Sierra Mojada No. 950, Col. Independencia, Guadalajara 44340, Mexico; carlos.mornelas@alumnos.udg.mx; 3 Instituto en Investigación en Ciencias Biomédicas, Centro Universitario de Ciencias de la Salud, Universidad de Guadalajara, Guadalajara 44340, Mexico; biologiamolecular@hotmail.com (J.F.M.-V.); chagollan@academicos.udg.mx (M.G.-C.); alexis.vizcaino@cucs.udg.mx (A.M.V.-Q.); 4 Laboratorio de Investigación en Cáncer e Infecciones, Departamento de Microbiología y Patología, Centro Universitario de Ciencias de la Salud, Universidad de Guadalajara, Guadalajara 44340, Mexico; daniel.diaz@academicos.udg.mx; 5 Doctorado en Psicología de la Salud (Modalidad Directa), Departamento de Psicología Básica, Centro Universitario de Ciencias de la Salud, Sierra Mojada No. 950, Col. Independencia, Guadalajara 44340, Mexico

**Keywords:** furin, viral pathogenesis, host–virus interactions, genetic susceptibility

## Abstract

Furin, a calcium-dependent serine endoprotease of the proprotein convertase family, plays a pivotal role in both physiological homeostasis and viral pathogenesis. By cleaving polybasic motifs within viral glycoproteins, furin enables the maturation of structural proteins essential for viral entry, fusion, and replication. This mechanism has been documented across a broad spectrum of human pathogens, including SARS-CoV-2, influenza virus, human immunodeficiency virus, human papilloma virus, hepatitis B virus, flaviviruses, herpesviruses, and paramyxoviruses, highlighting furin as a conserved molecular hub in host–virus interactions. Genetic variability within the FURIN gene further modulates infection outcomes. Several single-nucleotide polymorphisms (SNPs), such as rs6226 and rs1981458, are associated with altered COVID-19 severity, whereas variants like rs17514846 confer protection against human papilloma virus infection. Conversely, mutations predicted to reduce enzymatic activity have been linked to attenuated SARS-CoV-2 pathogenesis in certain populations. These findings underscore the importance of considering population genetics when evaluating viral susceptibility and disease progression. Despite advances, unresolved questions remain regarding furin’s non-canonical roles in viral life cycles, tissue-specific regulation, and interactions with other host proteases and immune modulators. Targeted inhibition of furin and related convertases represents a promising avenue for broad-spectrum antiviral interventions. Collectively, current evidence positions furin as a central node at the intersection of viral pathogenesis, host genetic variability, and translational therapeutic potential.

## 1. Introduction

Many bioactive proteins produced by organisms are initially synthesized as inactive precursors that require endoproteolytic processing for maturation and activation. This mechanism is widespread among organisms and regulates the activity of various hormones, growth factors, cell receptors, cell adhesion molecules, and viral envelope proteins, among others [1]. The proteins, or enzymes, responsible for this proteolytic process—the degradation of proteins into peptides through the hydrolysis of peptide bonds—are known as proteases or peptidases. These can, in turn, be classified as endoproteases or exoproteases depending on their cleavage site [2].

Proteases are present in practically all forms of life and are very numerous and varied. The development of the MEROPS peptidase database enabled the establishment of a hierarchical classification based on the mechanisms of action and characteristics of each protease. Currently, this classification recognizes the following families of proteases: asparagine, cysteine, glutamine, serine, and threonine peptidases; metalloproteinases; mixed peptidases; and peptidases with an unknown mechanism of action [2,3]. Furin belongs to a group of seven proprotein convertases (PC) in mammals, namely PC1/PC3, PC2, PC4, PACE4, PC5/PC6, PC7/PC8/LPC/SPC7, and furin itself [4,5]. To standardize nomenclature, some authors refer to their genes as PCSK (proprotein convertase subtilisin/kexin), with furin designated as PCSK3 [6]. Furin is a calcium-dependent serine endoprotease, classified in the MEROPS peptidase database as clan SB, family S8, subfamily S8B (peptidase S08.071), and designated by the IUBMB with the number EC 3.4.21.75. This enzyme is classified within the subphylum Vertebrata, and its reference sequence corresponds to Homo sapiens (UniProt: P09958) [7]. This protein is predominantly localized in the trans-Golgi network (TGN). However, it has also been detected in the plasma membrane, endosomes, immature secretory granules, and the extracellular matrix of eukaryotic cells, reflecting its ubiquitous distribution and participation in numerous protein maturation processes [8,9,10].

Numerous mutations have been identified that lead to clinically significant phenotypes, including those affecting the cardiovascular system, metabolism, mental health, and infectious diseases. For example, increases in furin protease caused by the rs17514846 variant are associated with coronary heart disease [11]. Also, the rs4702 polymorphism, an eQTL SNP for FURIN, in the homozygous state is associated with elevated systolic and diastolic blood pressure [12]. Moreover, reduced levels of furin expression may be linked to prediabetes, type 2 diabetes, and obesity [13]. Due to variants such as rs148110342, which have been related to high enzymatic activity, they are significantly associated with type 2 diabetes and changes in LDL cholesterol levels in Kawaii populations [14], and hypermethylation in two of the eight CpG sites tested in the FURIN promoter was associated with an increased risk of diabetes in Chinese populations in Gusu [15].

In other important clinical implications, the rs4702-A allele was associated with higher expression of FURIN and BDNF in the serum of glioma patients after radiotherapy, implying a lower risk of radiotherapy-induced cognitive impairment [16], and the negative regulation of FURIN expression by miR-338-3p, specific to the rs4702 G allele, is associated with schizophrenia [17].

Moreover, the involvement of furin in viral infections was also established. This finding was based on prior knowledge that the hemagglutinin (HA) of influenza viruses requires proteolytic processing to acquire infectivity [18]. In 1992, studies demonstrated that furin was responsible for cleaving and activating HA from the fowl plague virus (FVP) [18]. Subsequently, furin was also identified as responsible for activating the gp160 glycoprotein of the human immunodeficiency virus (HIV), which is crucial for membrane fusion [19]. Based on these fundamental findings, numerous studies have documented furin’s involvement in the life cycles of various viral pathogens [20]. In this article, we review the structural and functional characteristics of furin, as well as current findings on the relationship between furin mutations and the development and progression of viral diseases.

## 2. History of the Discovery of Furin

In 1947, Linderstrøm-Lang and Ottesen accidentally discovered an enzyme capable of modifying the structure of ovalbumin without degrading it. This enzyme was obtained from the bacterium Bacillus subtilis, which is why it would later be known as subtilisin [21]. After this discovery, researchers began to work on the methods for the isolation and crystallization of subtilisin [22,23], and it was not until 1966 that the complete amino acid sequence that comprised subtilisin was deciphered [24]. Finally, in 1969, the molecular structure of subtilisin was revealed at a 2.5 Å resolution [25].

In 1967, Steiner et al. demonstrated that insulin is synthesized as a high-molecular-weight protein, which they named proinsulin, and requires a post-translational process to acquire its bioactive form [26]. Chance et al. (1968) determined the amino acid sequence of swine proinsulin, identifying two cleavage sites (PQKR63↓ and AERR31↓) essential for the generation of mature insulin and C-peptide [27].

On the other hand, Chrétien & Choh (1967) conducted a comparative analysis of amino acid sequences of the hormones β-lipotropin (β-LPH), γ-lipotropin (γ-LPH), and β-melanotropin (β-MSH). They found that both β-MSH and γ-LPH corresponded to fragments contained within the β-LPH sequence and identified the cleavage motifs AEKK40↓ and KDKR60↓ as responsible for the release of these hormones [28]. Based on these findings, an increasing number of initially inactive proteins that require proteolysis at basic amino acids have been documented with acid-rich sites [29].

Between 1969 and 1970, Kraut et al. characterized the catalytic site of subtilisin BPN (an extracellular protease produced by *Bacillus subtilis*) after resolving its three-dimensional structure. This work revealed the existence of a catalytic triad composed of Asp32, His64, and Ser221, interconnected by hydrogen bonds that are essential for its proteolytic activity [30]. Today, subtilisin is one of the most studied proteases. It is classified as a serine protease and gave its name to one of the largest families of proteases, the subtilases (Subtilisin-like serine proteases). Serine proteases have as their main characteristic a catalytic center formed by three amino acids: Asparagine, Histidine, and Serine; however, the amino acid that carries out the nucleophilic attack is serine, which is why they acquire their name of serine proteases [2,31,32].

Most of the studied subtilases so far were those belonging to microorganisms such as bacteria and yeasts. In 1976, Leibowitz et al. demonstrated that mutations in the *Kex2* gene of the yeast *Saccharomyces cerevisiae* prevented the production of alpha-factor and killer toxins, both of which are dependent on cleavage at basic amino acid sites for activation [33,34]. Subsequent studies established that kexin, the enzyme encoded by the Kex2 gene, is an endoprotease with a catalytic site analogous to that of bacterial subtilisins (Asp, His, and Ser), capable of hydrolyzing dibasic motifs such as -Lys-Arg- (KR↓) and -Arg-Arg- (RR↓) with similar efficiency, and whose activity depends on Ca^2+^ ions [34].

Despite their origin in distinct evolutionary domains (bacteria in the case of subtilisin and eukaryotes such as yeast for *kexin*), their structural and functional similarities led to the classification of *kexin* as a member of a subtilisin subfamily [35,36]. Furthermore, between 1987 and 1988, studies demonstrated that *kexin* could process both pro-albumin and proopiomelanocortin (POMC) in mammals [37,38]. These findings supported the hypothesis that *Kex2* represented the prototype of a family of homologous convertase enzymes in mammals, which motivated the search for such homologs. This group of homologous enzymes was named proprotein/prohormone convertases [36].

**Figure 1:**
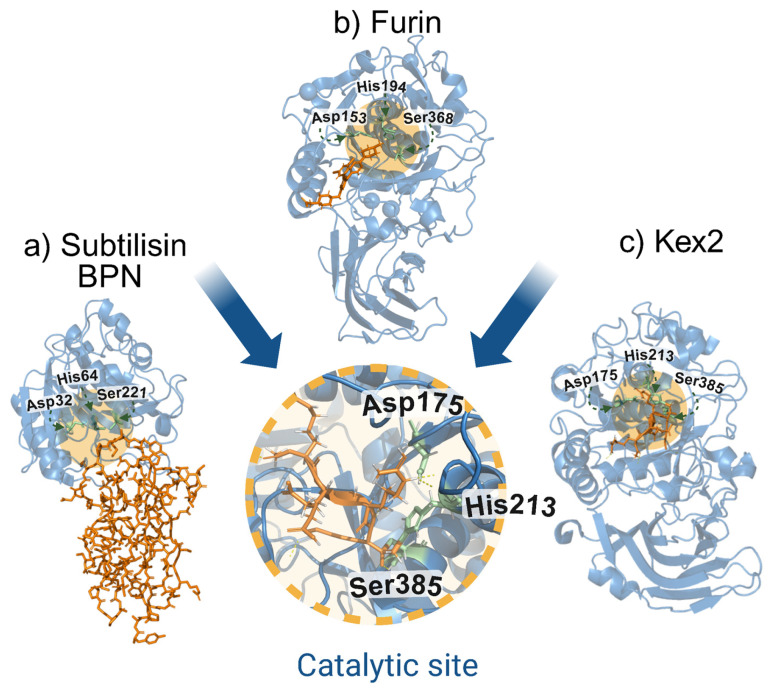
Common catalytic site among subtilisin-like serine proteases: subtilisin BPN, furin, and Kex2. (**a**) Structure of the complex of subtilisin BPN and Streptomyces subtilisin inhibitor (SSI), (PDB: 2SIC). (**b**) Structure of human furin bound to BOS-318, a small-molecule peptidomimetic inhibitor targeting its active site (PDB: 2ID4). (**c**) Crystal structure of yeast Kex2 protease in complex with a peptidyl-chloromethylketone inhibitor (PDB: 2ID4). The central inset highlights the conserved catalytic triad (Asp–His–Ser) shared among all three enzymes, characteristic of the subtilisin-like serine protease family.

Furin was one of the first proprotein convertases (PCs) to be identified [36]. In 1986, Roebroek et al. described a region located upstream of the proto-oncogene FES/FPS on human chromosome 15, which they named FUR (FES/FPS Upstream Region). Its putative protein product was designated furin [39]. Initially, its function remained unknown, and it was not considered a PC; in fact, members of the same research group proposed furin as a marker for small-cell and non-small-cell lung carcinomas in 1987, finding elevated mRNA levels in the liver and kidney associated with these pathologies [40]. A decisive breakthrough occurred in 1989, when Fuller et al. conducted a comparative analysis of amino acid sequences, revealing that furin shared 50% identity with the C-terminal one-third of the catalytic domain of *Kex2*. This study identified key residues (such as serine and asparagine) conserved in subtilisin-like proteases, suggesting its potential as a proprotein-processing enzyme [41]. This hypothesis was reinforced in 1990 by Van den Ouweland et al., who fully sequenced the FUR gene and confirmed its homology with the kexin of *Saccharomyces cerevisiae* [42]. During that same year, experimental evidence validated the role of furin as a proprotein convertase: assays demonstrated its ability to cleave the proforms of beta-nerve growth factor (pro-β-NGF), a neurotrophin critical for neuronal survival and differentiation, as well as the pro-form of von Willebrand factor (pro-vWF), a key glycoprotein involved in primary hemostasis and blood coagulation [43,44]. Its ability to recognize and cleave dibasic motifs in human substrates, especially in the processing of pro-vWF, led to furin being alternatively called PACE (Paired Basic Amino Acid Cleaving Enzyme) [45] beside proprotein convertase subtilisin/kexins (PCSKs), a nomenclature based on their human genes [8]. Nowadays, furin is one of the most widely studied proteases in mammals [1,46]. Figure 1 shows the structure of the catalytic domain from furin compared to other related proteases [45].

## 3. Furin

### 3.1. Encoding

As previously described, furin is encoded by the *FUR* gene, located between positions 90,868,588 and 90,883,458 in the human genome (GRCh38/hg38). This region spans 14,871 base pairs and encodes the furin protein [47]. The expression of this gene is regulated by a system of three alternative promoters: P1A, P1B, and P1. The P1A and P1B promoters, which are characterized by high GC content and the absence of typical TATA and CAAT regulatory boxes, contain multiple binding sites for the transcription factor Sp-1, suggesting constitutive gene expression in most tissues and cell types. In contrast, the P1 promoter exhibits a more complex regulatory profile, integrating canonical elements such as TATA and CAAT boxes along with binding sites for specific transcription factors, including C/EBPβ, Smad2/4, GATA-1, and HIF-1. This configuration enables fine-tuned regulation of gene expression in response to cellular signals [48,49,50,51].

### 3.2. Molecular Structure and Functional Domains of Furin

Furin is a type I transmembrane protein composed of 794 amino acids, whose structure organizes its domains in sequential order: a signal peptide (1–26 aa), a prosegment (27–107 aa) containing two cleavage sites RX(R/K)R↓, a subtilisin-like catalytic domain (108–435 aa) with the characteristic triad Asp153-His194-Ser368 (DHS), a P or homoB domain (436–576 aa), a cysteine-rich domain (577–714 aa), a transmembrane helix (TM) (715–738 aa), and a cytoplasmic tail (739–794 aa) [47,52,53]. As a type I transmembrane protein, it displays a characteristic topological orientation, with the N-terminal domain extending into the lumen of an organelle or to the extracellular space, and the cytoplasmic C-terminal tail localized within the cytoplasm [54,55].

Within the P domain (homoB), the Arg498-Gly499-Asp500 motif (referred to as the RGD motif) plays a crucial role in activity by potentially facilitating interaction with integrins [47,56]. Additionally, three calcium-binding sites (Ca^2+^) regulate its structural conformation and function, displaying different affinities: Ca^2+^ (I) has a higher affinity for furin than Ca^2+^ (II), which in turn is higher than Ca^2+^ (III) [57]. Each site is associated with specific amino acids: Ca^2+^ (I) is Asp115-Asp162-Val205-Asn208-Val210-Gly212, Ca^2+^ (II) is Asp-Asp301-Glu331, and Ca^2+^ (III) is Asp174-Asp179-Asp181 [57]. The calcium dependency of furin can be observed in the oxyanion hole, where Asn295 (N295), although not part of the catalytic triad, undergoes a slight conformational change after binding Ca^2+^ (II). This enables N295 to form a hydrogen bond with the carbonyl oxygen of the peptide bond to be cleaved, thereby polarizing it and facilitating bond cleavage [57,58].

**Figure 2:**
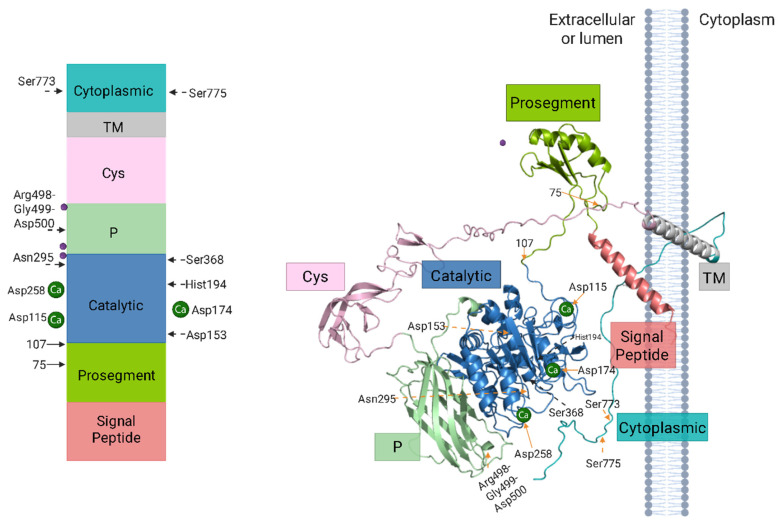
Structural organization of preprofurin (AlphaFold ID AF-P09958-F1). The schematic representation illustrates the domain architecture of preprofurin, with distinct functional regions color-coded: signal peptide (coral), propeptide (green), catalytic domain (blue), P-domain/homoB (light green), cysteine-rich region (pink), transmembrane domain (gray), and cytoplasmic domain (aqua green). Key structural and functional elements are annotated: phosphorylation sites (Ser773-Ser775), the RGD motif within the P-domain (Arg498-Gly499-Asp500), catalytic triad (Asp153-His194-Ser368) with the oxyanion hole (Asn295), and N-glycosylation sites (purple circles at Asn387, Asn440, and Asn553). Autocatalytic cleavage sites are indicated (R75↓ and R107↓). Calcium ion binding sites are indicated by the primary interacting residues (Asp115, Asp174, Asp258). This comprehensive visualization integrates structural predictions from AlphaFold with experimentally validated functional motifs. Figure Modified from Osman et al. (2022) [47].

On the other hand, furin possesses three potential N-glycosylation sites (Asn387, Asn440, and Asn553) essential for proper maturation and activation of the enzyme [8,56]. Lastly, furin contains two post-translational phosphorylation sites, Ser773 and Ser775. This modification, catalyzed by casein kinase II (CKII), together with a cluster of acidic amino acids (mainly Asp and Glu), facilitates furin’s interaction with components of the cellular trafficking system and ensures its localization in the TGN (Figure 2) [59].

### 3.3. Mechanisms of Furin Activation and Maturation

**Figure 3:**
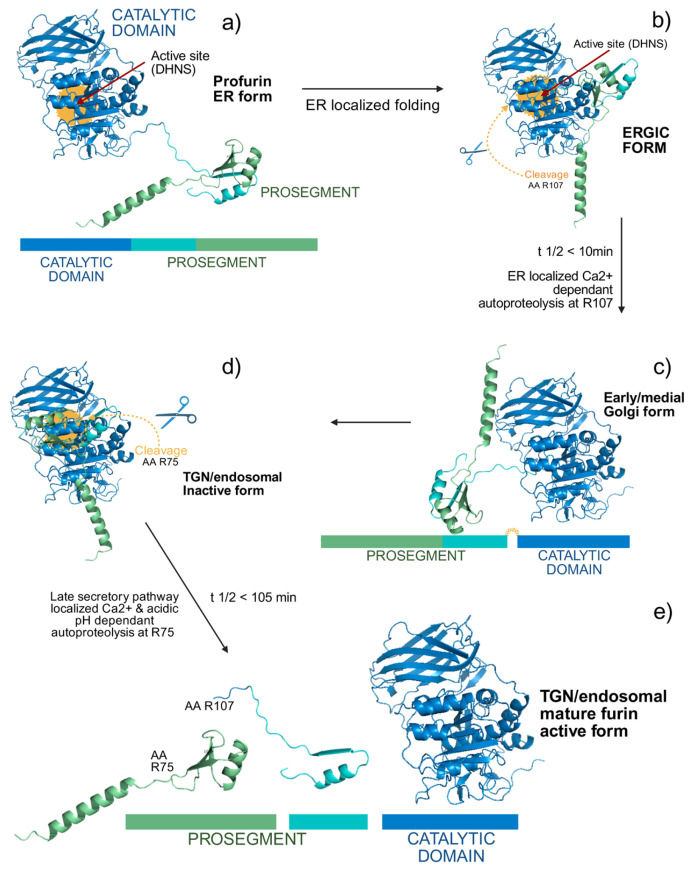
Schematic representation of the furin activation pathway. The maturation process of furin is depicted in four sequential stages: (**a**) full-length profurin containing the inhibitory propeptide domain; (**b**) after proper folding in the endoplasmic reticulum (ER), profurin undergoes initial autocatalytic cleavage at R107↓. The liberated propeptide remains non-covalently associated as an inhibitor, leading to transient accumulation within the ER-Golgi intermediate compartment (ERGIC) and cis-Golgi network (CGN); (**c**) subsequent transport to late secretory compartments (trans-Golgi network [TGN] or endosomes) facilitates the second autocatalytic processing event at R75↓, which occurs under specific environmental conditions, including acidic pH and elevated calcium concentrations; (**d**) the final dissociation of inhibitory propeptide fragments yields the fully active furin protease, capable of substrate recognition and cleavage (e) giving a final and active form of the protein. Figure Modified from Thomas et al. (2002) Preprofurin (UniProt: AF-P09958-F1) [55].

Furin initially exists as an inactive precursor (profurin, with a molecular weight of ap-proximately 100 kDa) and becomes active through a two-step autocatalytic process. The autolytic cleavage results in the formation of an active 90 kDa enzyme, characterized by N-terminal truncation [52,53,60]. After being synthesized in the endoplasmic reticulum (ER), furin begins its activation process as an inactive zymogen. During the initial stage, the signal peptide is removed, and the proenzyme undergoes N-glycosylation at multiple sites, modifications that are crucial for proper folding in the ER. Because of the acidic pH and reductive environment within the ER, furin employs its intrinsic catalytic activity to initiate maturation. This occurs via an autocatalytic cleavage event at the RTKR107↓ motif, delineating the boundary between its catalytic domain and the prosegment region (Figure 3).

This preliminary processing event, characterized by an approximate half-life of 10 min, is critical for the translocation of the enzyme to the Golgi apparatus. After this initial cleavage, the liberated prosegment remains non-covalently associated with the catalytic domain, thereby maintaining furin in an inactive conformation. Full activation is only achieved when the enzyme reaches the TGN, where acidic conditions and elevated Ca^2+^ concentrations favor a second autocatalytic cleavage event at the RGVTKR75↓ site. This second event, characterized by a half-life of approximately 105 min, induces a conformational change that releases the prosegment and yields the mature, active form of the enzyme. This regulated control ensures that furin acquires its catalytic activity in the appropriate cellular compartments, preventing premature degradation of its substrates [8,61,62,63].

### 3.4. Intracellular Trafficking Dynamics and Recycling of Furin

Once activated in the TGN, furin is distributed to various cellular compartments through a complex recycling system, which is described below. In these locations, furin is active and processes its substrates optimally under specific conditions, which include a pH range of 7 to 7.5. Nevertheless, the enzyme maintains more than 50% of its maximum activity within a pH range of 6 to 8.5 and calcium concentrations from 0.2 to 10 mM [9,38,40,64]. The Golgi apparatus, composed of multiple stacked cisternae interconnected by an extensive tubular-reticular network, comprises two primary functional regions: the cis-Golgi network (CGN), positioned proximal to the endoplasmic reticulum-Golgi intermediate compartment (ERGIC), and the TGN [65]. Furin follows this complex intracellular trafficking route that originates in the TGN, regulated by defined signaling motifs in its 56-amino-acid cytosolic domain (Figure 2). This region mediates its participation in two recycling pathways: (1) TGN–endosomes and (2) plasma membrane–early endosomes [66].

During the initial cycle, furin is transported from the TGN to endosomes, a process mediated by hydrophobic sorting motifs that contain tyrosine or dileucine-like sequences. These motifs facilitate interaction with clathrin-associated adaptor proteins, such as AP-1, which directs furin toward endosomes or immature secretory granules (ISGs), and AP-4, which mediates its transport to the basolateral surface. The return of furin to the TGN from these compartments is mediated by Phosphofurin Acidic Cluster Sorting protein-1 (PACS-1), which specifically recognizes the acidic cluster (AC) of furin after its phosphorylation by casein kinase 2 (CK2). This CK2/PACS-1-dependent process also applies to the return of furin from ISGs to the TGN.

During the second cycle, furins that reach the plasma membrane can transiently anchor to it via interaction with filamin ABP-280. This cytoskeletal protein organizes orthogonal actin networks and connects surface receptors with the subcortical scaffold. Alternatively, furin can be internalized via clathrin- and dynamin-dependent endocytosis, mediated by AP-2, which recognizes tyrosine-based motifs. In the early endosomes, furin phosphorylated by CK2 can be recycled back to the plasma membrane in a PACS-1-dependent step [55,66].

**Figure 4:**
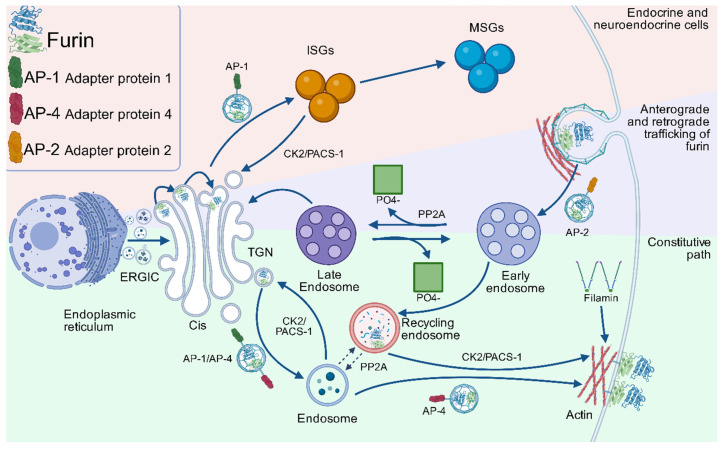
Intracellular trafficking pathways of furin in different cellular compartments. The schematic illustrates three distinct trafficking routes of furin, each color-coded to represent specific cellular processes: The red pathway depicts endocrine/neuroendocrine cell-specific trafficking, where AP-1 mediates furin budding from the trans-Golgi network (TGN) to immature secretory granules (ISGs). In this pathway, phosphofurin acidic cluster sorting protein 1 (PACS-1) functions as an adaptor, linking CK2-phosphorylated furin to the AP-1/clathrin machinery for TGN retrieval. Alternatively, furin-containing vesicles may progress to mature secretory granules (MSGs) for hormone processing. The green pathway represents basolateral targeting, where AP-4 directs furin transport from either the TGN or endosomal compartments to the basolateral membrane. At the membrane, filamin (ABP-280) anchors furin to the actin cytoskeletal network. The blue pathway encompasses complex bidirectional trafficking between multiple compartments. Furin internalization from the plasma membrane occurs via clathrin and dynamin-dependent endocytosis, primarily mediated by tyrosine-based motifs interacting with AP-2. During endosomal transit, PX domain-containing phosphoinositide-binding proteins modulate furin sorting. Early endosomes serve as decision points (1). PP2A-mediated dephosphorylation targets furin for TGN return via late endosomal intermediates, with phosphate release (2). CK2-phosphorylated furin undergoes PACS-1-dependent recycling to the plasma membrane. Dashed lines indicate the putative PP2A-dependent route from post-TGN endosomal compartments to the cell surface. Throughout these pathways, CK2 and PACS-1 participate at multiple trafficking nodes, highlighting their central role in furin localization dynamics. This integrated model demonstrates how post-translational modifications (phosphorylation and dephosphorylation) together with adaptor protein interactions coordinate furin’s subcellular distribution and functional specialization across different cell types. Figure Modified from Thomas et al. (2002) [55].

Both cycles are interconnected when furin is redirected from early endosomes to the TGN after dephosphorylation of its acidic cluster by Protein Phosphatase 2A (PP2A) isoforms containing B-family regulatory subunits. This same phosphatase is also responsible for furin trafficking from post-TGN endosomes to the cell surface (Figure 4) [55,66]. This recycling system ensures the distribution of furin among subcellular compartments, allowing it to participate in both constitutive and regulated secretion processes [55].

## 4. Functional Diversity of Furin Substrates

As mentioned above, PCs share the ability to cleave proproteins at motifs (Arg/Lys)Xn(Arg/Lys)↓ (abbreviated (R/K)Xn(R/K)↓), where “n” can be 0, 2, 4, or 6 [47]. Furin exhibits a preference for RXn(R/K)R↓ sequences, with cleavage occurring at the C-terminal end of the motif [20]. Furin’s selectivity for basic residues (arginine/lysine) is attributed to the architecture of its active site. The substrate-binding pockets are lined with negatively charged amino acid residues (e.g., Asp/Glu), which establish electrostatic interactions with the guanidinium (Arg) or amino (Lys) groups of its substrates. This charge-charge complementarity determines its affinity for motifs rich in basic amino acids [7,67]. Unlike other PCs, furin exhibits a ubiquitous expression pattern in humans, as demonstrated by the widespread presence of its mRNA across all organ systems. This broad distribution suggests its involvement in the processing and activation of numerous protein precursors [20]. To date, more than 400 substrates have been identified as containing the characteristic consensus motif recognized by furin, and these are functionally classified into several major groups [47]. Among them are plasma proteins such as albumin [68,69], complement C3 [69], coagulation factors IX and X [70,71], von Willebrand factor (vWF) [45,72,73], and protein C [74]. In the endocrine system, furin is involved in the maturation of various peptide hormones, including parathyroid hormone (PTH) [75], its homolog PTHrP [76], and growth hormone-releasing hormone (GHRH) [77], as well as neurotrophic factors such as brain-derived neurotrophic factor (BDNF) and neurotrophin-3 (NT3) [78].

Additionally, the enzyme plays a particularly important role in activating components of the cell signaling system, processing both ligands and membrane receptors. Among the ligands are endothelin-1 (ET1) [79], anti-Müllerian hormone (AMH) [80], insulin-like growth factor I (IGF-I) [81], transforming growth factor β1 (TGFβ1) [82], and vascular endothelial growth factors D (VEGF-D) and C (VEGF-C) [83,84]. It also enables the activation of receptors such as the insulin receptor [85], the hepatocyte growth factor receptor [86], the low-density lipoprotein receptor-related protein [87], integrins α3, α6, and αv [88], and the Notch-1 receptor [89]. Some extracellular matrix metalloproteinases, such as MMP1 [90], stromelysin-3 [91], ADAM19 [92], ADAMTS-1 [93], and BMP-1 [94] are also processed. Other extracellular matrix components include collagen (e.g., type 23) [95] and fibrillin (e.g., type 1) [96].

**Figure 5:**
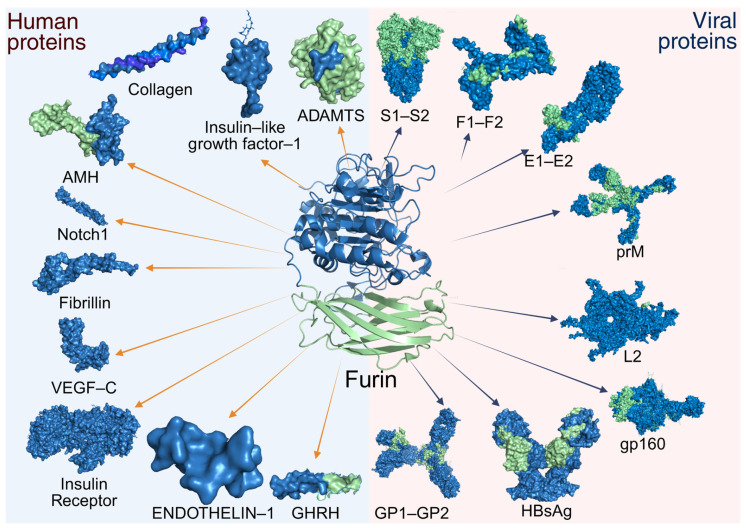
Comparative analysis of furin-mediated proteolytic processing in viral and human proteins. The schematic depicts the conserved role of furin in the proteolytic maturation of key viral structural proteins across different pathogen families, alongside representative human substrates. Highlighted viral targets include the SARS-CoV-2 spike (S) protein (PDB: 8T21; family Coronaviridae), the measles virus fusion protein F0 (PDzB: 5YXW; Paramyxoviridae), the chikungunya virus E1–E2 precursor (PDB: 3N42; Togaviridae), the dengue virus prM protein (PDB: 8FE3; Flaviviridae), the human papillomavirus L2 capsid protein (PDB: 5KEP; Papillomaviridae), the HIV gp160 envelope precursor (PDB: 6PWU; Retroviridae), the hepatitis B virus HBsAg surface antigen (PDB: 3V6Z; Hepadnaviridae), and the Ebola virus GP1–GP2 glycoprotein precursor (PDB: 3CSY; Filoviridae). The diagram emphasizes evolutionary convergence on furin-mediated processing as a critical maturation step for diverse viral envelopes and capsid proteins, while also indicating relevant human physiological substrates for comparative context. This conservation across phylogenetically distinct viruses underscores the central importance of host proprotein convertases in viral pathogenesis and highlights potential therapeutic targets for the development of broad-spectrum antivirals. Additionally, several human proteins are matured by furin, including ADAMTS-4 (PDB: 4WK7), insulin-like growth factor-1 (PDB: 1IMX), collagen (PDB: 6A0C), anti-Müllerian hormone (AMH; PDB: 7L0J), Notch1 (PDB: 4XL1), fibrillin-1 (PDB: 5MS9), VEGF-C (PDB: 4BSK), the insulin receptor (PDB: 6CE9), endothelin-1 (PDB: 1EDN), growth hormone-releasing hormone (GHRH; PDB: 7CZ50 and Furin (PDB: 5JXG).

These elements are essential for the proper functioning and homeostasis of the human body. However, furin has also been potentially linked to pathophysiological processes, such as neurodegenerative or neuropsychiatric disorders (e.g., Alzheimer’s, schizophrenia, epilepsy, or ischemia) [97], type 2 diabetes mellitus [13], arterial hypertension [98], the development of atherosclerosis and cardiovascular diseases [6], the progression of tumors or cancer (carcinomas, sarcomas, skin cancer, as well as brain or central nervous system tumors) [99], and the activation of bacterial toxins (e.g., anthrax, diphtheria, Pseudomonas, and Shiga toxins), this proetolytic process is crucial for the separation of A subunit from the membrane-associated B subunit, which allows it to exert the toxic effect [100,101,102,103,104]. In addition to its role in physiological processes, furin also participates in the activation of viral glycoproteins, linking it to the pathogenesis of a wide range of viral infections (Figure 5) [20].

## 5. Furin-Dependent Viral Processing Mechanisms

### 5.1. Human Papillomavirus

Human papillomavirus (HPV), a non-enveloped, double-stranded DNA virus of the Papillomaviridae family, encompasses over 100 genotypes stratified by their oncogenic risk [105]. HPV exhibits a specific tropism for basal epithelial cells, where it establishes persistent infections [106,107]. Its icosahedral capsid, composed of the major (L1) and minor (L2) structural proteins, mediates direct interaction with host cells. Furin plays a critical role in the infectious cycle by cleaving the L2 protein, an essential step for viral entry [108].

The initial attachment to heparan sulfate proteoglycans (HSPGs), primarily via L1, triggers conformational changes in the capsid. This binding exposes the N-terminal region of L2, unveiling a highly conserved furin cleavage site at Arg12 [109,110]. While other cleavage motifs exist in L2, proteolysis at Arg12 is the event consistently linked to productive infection [110]. This furin-mediated cleavage exposes critical domains on the capsid that are necessary for internalization via a secondary receptor and endocytosis [109,111]. It is important to note that although L1 can also be proteolytically processed, this is not furin-dependent and its role in infectivity remains unclear, potentially relating to viral morphogenesis [112]. Thus, L2 cleavage at Arg12 stands as the sole, indispensable furin-dependent event for HPV infectivity [112].

The efficiency of this processing step is mainly dependent on two key factors. First, as cleavage occurs at the plasma membrane prior to internalization, it is highly dependent on local furin availability. In vitro studies corroborate this, showing that only 25–34% of L2 is typically cleaved within 24 h, which can rise to 55% with furin overexpression and correlates with a 5- to 6-fold increase in infectivity [109]. Second, there is significant genotypic variability in furin sensitivity. Genotypes such as 6, 16, 45, 52, and 58 are highly dependent, whereas types 18 and 31 are largely resistant to furin inhibition, implying the existence of alternative activation mechanisms [113].

### 5.2. Influenza Virus

**Figure 6:**
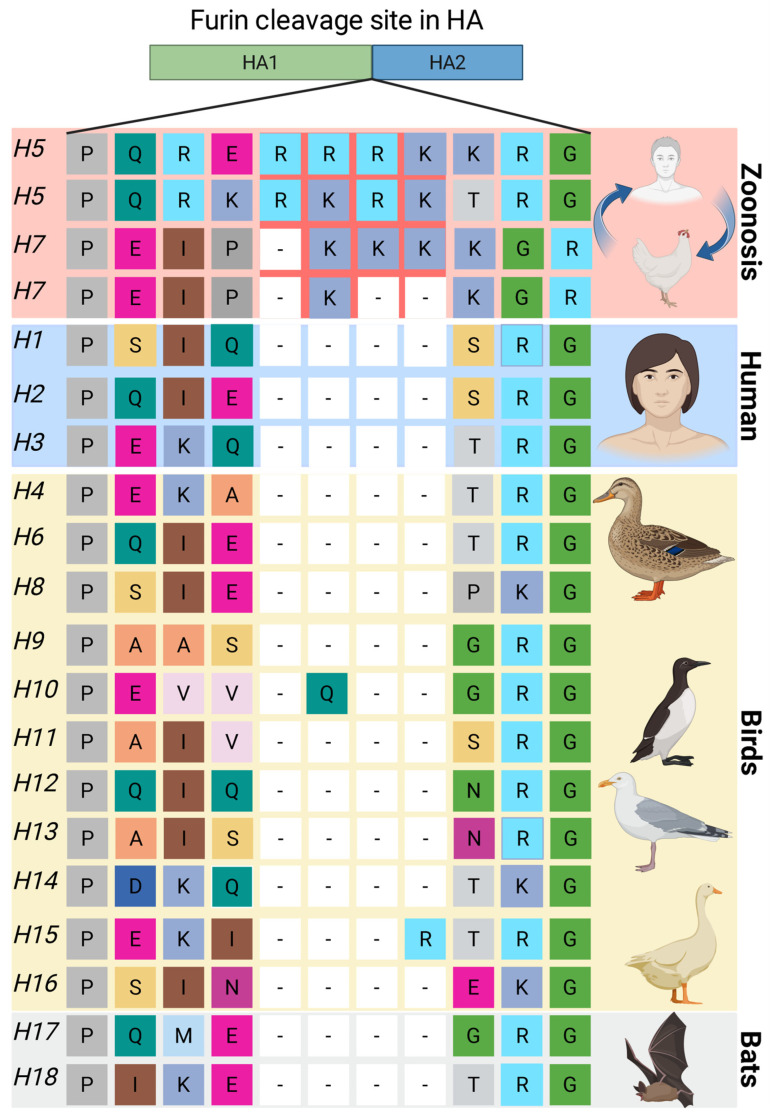
Comparative alignment of influenza hemagglutinin cleavage sites. The multiple sequence alignment depicts the evolutionary divergence in hemagglutinin (HA) cleavage site motifs among influenza A virus subtypes. Human-adapted subtypes (H1, H2, and H3) typically possess monobasic cleavage sites. In contrast, the highly pathogenic avian influenza subtypes H5 and H7 may harbor a polybasic cleavage motifs that enable furin-mediated proteolytic activation, significantly enhancing viral pathogenicity. Notably, subtypes H4 through H18 (excluding H5 and H7) consistently lack polybasic cleavage sites, maintaining the ancestral monobasic configuration. The alignment, generated using CLUSTAL Omega v. 1.2.2, includes representative sequences from Spanish influenza H1N1 (AAD17229.1); H2N9 (ADU17660.1); seasonal H3N2 (ABD59856.1); avian H4N6 (AHL82211.1), H5N1 (AAD13569.1), H5N2 (AAC58990.1), H6N8 (AKF34349.1), H7N7, (AAC54377.1), H7N? (AAC54381.1) H8N4 (CAY39405.1), H9N2 (AAV68030.1), H10N7 (ADP07130.1), H11N2 (AHZ21120.1), H12N6 (AGG84126.1), H13N9 (ADB46159.1), H14N3 (AHJ57334.1), H15N2 (ABB90704.1), H16N3 (BAO94331.1); bat-derived H17N10 (AFC35438.1): and H18N11 (AKC43903.1). This comprehensive phylogenetic comparison highlights the exceptional nature of polybasic cleavage site acquisition in H5 and H7 subtypes, a key molecular determinant of influenza virulence and host range.

Influenza A virus, a segmented RNA member of the Orthomyxoviridae family, is a highly contagious respiratory pathogen. Its classification is based on two surface glycoproteins, hemagglutinin (HA; 18 subtypes) and neuraminidase (NA; 11 subtypes), with the proteolytic activation of HA being a critical determinant of infectivity and tropism [114,115]. Viral entry absolutely requires the cleavage of the 322–329 region of the precursor hemagglutinin (HA0) into the subunits HA1 and HA2, this cleavage liberates the hydrophobic fusion peptide located at the N-terminus of HA2, a step that primes the protein for receptor binding and catalyzes membrane fusion [114,115,116,117]. The cleavage site resides in a surface-exposed loop of HA0 and is a key molecular determinant of pathogenicity. These sites are categorized as either monobasic or multibasic [118]. Multibasic cleavage sites, a hallmark of highly pathogenic avian influenza (HPAI) strains, are efficiently recognized by ubiquitous proprotein convertases, such as furin. This allows for systemic infection beyond the respiratory tract, leading to severe pathogenicity. In contrast, human-adapted influenza strains possess monobasic sites, which are cleaved only by trypsin-like proteases found in specific tissues (e.g., the respiratory tract), thereby restricting their tropism and typically resulting in less severe, localized infections (Figure 6) [114,115].

Consequently, the role of furin in the lifecycle of established human seasonal influenza viruses is minimal, as they uniformly lack multibasic motifs [119]. This is corroborated by protease inhibitor studies, which show that infection by these strains relies exclusively on trypsin-like proteases [120]. However, furin remains a critical factor in the emergence of potential pandemic strains. Research on zoonotic viruses, such as the H9N2 strain (Israel810, motif R-S-K-R), demonstrates that furin cleavage efficiency is directly proportional to cellular furin levels [121]. Furthermore, specific post-translational modifications in HA, like the loss of a glycosylation site at position 13—a mutation observed in natural isolates—can significantly enhance cleavage by endogenous furin, even at suboptimal motifs [121].

In summary, furin’s role in influenza pathogenesis is primarily restricted to: (1) viruses possessing multibasic cleavage sites, which are almost exclusively avian-derived variants with high zoonotic and pandemic potential [116,119], and (2) specific cellular contexts that favor furin activity, such as its overexpression or permissive HA modifications [121]. This strict selectivity underscores the fundamental role of the viral HA cleavage site-host protease compatibility in governing tissue tropism and ultimate pathogenicity.

### 5.3. Herpesviridae Family

The Herpesviridae family comprises enveloped, double-stranded DNA viruses, taxonomically divided into the subfamilies Alphaherpesvirinae, Betaherpesvirinae, and Gammaherpesvirinae [122]. These viruses exhibit characteristic tropism for epithelial and neural tissues, which underpins their ability to establish lifelong latent infections [123,124]. Structurally, herpesviruses possess an icosahedral capsid surrounded by a lipid envelope that contains numerous integral glycoproteins forming a spike network on the surface, which is essential for mediating virus–host interactions, including attachment and entry into target cells [125].

Among these glycoproteins, the glycoprotein B (gB) is highly conserved across the herpesvirus family and plays a fundamental role in membrane fusion during viral entry. To acquire fusogenic activity, gB is synthesized as an inactive precursor (pro-gB) that requires proteolytic cleavage. This critical maturation step is catalyzed by host proprotein convertases, with furin as the primary enzyme, and occurs during transit through the secretory pathway, specifically within the trans-Golgi network (TGN) [126,127,128,129]. While furin is the dominant convertase, other PCs such as PC5/6 and PACE4 can also process gB in certain cellular contexts, highlighting a degree of redundancy in this essential activation step [128].

The functional significance of furin-mediated gB processing has been rigorously demonstrated in several of the eight known human herpesviruses. In Epstein–Barr virus (EBV), a deletion mutant lacking the RXK/RR cleavage motif (gB Δfurin) exhibited a severe fusogenic deficit, retaining only 48% and 72% of the wild-type fusion activity in epithelial cells and B lymphocytes, respectively [130,131]. A parallel requirement for furin cleavage was confirmed in varicella-zoster virus (VZV), where mutants with altered cleavage motifs (Δ491RSRR494 and 491GSGG494) showed attenuated replication in human skin xenografts, directly linking the integrity of the furin site to viral pathogenicity in vivo [132]. Robust evidence also exists for the role of furin in the maturation of gB in human cytomegalovirus (HCMV) and herpes simplex virus type 2 (HSV-2) [133,134,135]. Proteolytic cleavage is thought to trigger a conformational change in gB from a prefusion to a stable postfusion state, thereby activating its membrane fusion machinery and making it competent for driving the merger of the viral envelope with the host cell membrane [136].

These observations have positioned furin inhibition as a potential therapeutic strategy. Molecules such as α1-PDX, a general PC inhibitor, and naphtofluoroscein have been used as furin inhibitors [137], demonstrating an order of magnitude greater efficacy than current antiherpetic agents in cellular models. Its mechanism involves forming a stable complex with furin that is subsequently degraded, effectively blocking HCMV maturation and virus production [138,139]. These results underscore the central role of furin in the herpesvirus life cycle and validate its relevance as a target for antiviral development.

### 5.4. Togaviridae Family

Within the Togaviridae family, furin-dependent processing is a distinctive feature of the Alphavirus genus, with no such role documented for the Rubivirus genus (which includes rubella virus). Alphaviruses are spherical, enveloped viruses with a broad range encompassing mammals and birds and are primarily transmitted by arthropod vectors. Medically significant arthritogenic species, such as Chikungunya virus (CHIKV) and Semliki Forest virus (SFV), are notable for causing severe musculoskeletal disease [140].

The alphavirus genome is a single positive-sense RNA strand that encodes a polyprotein precursor for the structural proteins. This precursor is cleaved to produce the capsid protein (C) and the envelope glycoproteins E1, E2, E3, and 6K [140]. Initial cleavage events, mediated by viral or cellular proteases, release the individual proteins with one critical exception: the E2 and E3 glycoproteins remain linked as an E3-E2 precursor [141]. This precursor associates with E1 to form E3-E2-E1 heterotrimeric spikes on the virion surface [142]. The retention of E3 thus acts as a functional safety clip, preventing premature fusion in the acidic environment of the secretory pathway. Furin-mediated cleavage of E3 from E2 during virion transit through the TGN is the critical step that primes the spike for its fusogenic potential upon encountering the neutral pH of the extracellular environment or the acidic endosome of a new host cell [143,144,145].

While viral particles can be produced and secreted in the absence of furin cleavage, these virions are non-infectious [141,142,146,147]. Studies using furin-deficient cells or viruses with mutated cleavage sites confirm that the loss of E3-E2 processing abolishes infectivity and replicative capacity without impairing particle assembly or egress [141,142]. Structural analyses provide a mechanistic basis for this defect: immature virions with uncleaved E3-E2 possess a compact nucleocapsid that poorly exposes the genomic RNA, and their spike proteins fail to properly configure the E1 fusion loop, thereby crippling the membrane fusion machinery essential for entry [142].

The dependence on host convertases varies among alphavirus strains, influencing their biology and therapeutic susceptibility. For instance, CHIKV strains of African origin (with an HRQRR64 motif) are predominantly cleaved by furin and PC5B. In contrast, Asian lineage variants (featuring an RRQRR64 motif) are processed by a wider spectrum of proprotein convertases and consequently show reduced sensitivity to specific furin inhibitors like chloroquine [147]. Intriguingly, the inhibitory effects of chloroquine extend to mature virions, suggesting that furin activity may also be indirectly required for the proper maturation of cellular factors, such as receptors, that are necessary for a successful CHIKV infection [147]. This underscores a dual role for furin in alphavirus pathogenesis: enabling direct viral maturation through spike protein processing and potentially conditioning the host cell environment to support infection.

### 5.5. Hepatitis B Virus

Hepatitis B virus (HBV), the prototypical member of the Hepadnaviridae family, is an enveloped, hepatotropic pathogen with a partially double-stranded circular DNA genome. It is a major global cause of acute and chronic hepatitis, with outcomes ranging from spontaneous clearance to cirrhosis and hepatocellular carcinoma. The viral genome encodes three related envelope proteins (large-L, medium-M, and small-S, which constitute the surface antigen HBsAg) and the core protein (HBcAg), which forms the viral nucleocapsid. A key regulator of the host immune response is the hepatitis B e antigen (HBeAg), a secreted protein derived from the pre-core precursor that serves as a clinical marker of active replication and chronicity [148,149]. Following infection, HBV frequently integrates into the host genome, potentially facilitating sustained biosynthesis of viral components, even during suppressive antiviral therapy. This underscores the importance of understanding the maturation pathway of HBeAg, as its continuous presence fuels chronic liver disease [148,149].

Furin plays a critical role in the complex biosynthetic pathway of HBeAg. The pre-core precursor is directed to the endoplasmic reticulum by an N-terminal signal peptide, where initial processing yields an intermediate form known as pre-HBe or P22. This precursor is then trafficked to the TGN, where furin catalyzes the essential C-terminal cleavage event that generates the mature, secreted HBeAg [150,151,152]. Among six identified potential cleavage motifs, 151RXXR154 is the preferred furin site. Alternative processing can occur at the 164RRRR167 motif, producing a P20 intermediate that requires subsequent cleavage for final maturation [150]. This specificity is influenced by viral genotype; for example, genotype A harbors a DR insertion at the 151–154 motif, shifting furin processing to alternative sequences such as RRDR, RRGR, or RSPR [152].

The functional importance of this furin-dependent step extends beyond antigen secretion. Furin inhibition disrupts viral nucleocapsid stability, suggesting that HBeAg plays an underappreciated role in virion morphogenesis. This is supported by evidence that the unprocessed P22 precursor is misincorporated into nucleocapsids, leading to the production of unstable cores and a significant compromise in viral replication efficiency [153]. These findings position furin as a promising host-directed therapeutic target. Inhibiting furin, particularly in combination with conventional nucleos(t)ide analogs, could simultaneously disrupt multiple stages of the viral life cycle—including virion assembly and the production of a critical immunomodulatory protein—potentiating treatment efficacy [154]. A deeper understanding of these mechanisms not only clarifies fundamental HBV biology but also paves the way for innovative combination therapies against this pervasive infection.

### 5.6. Filoviridae Family

The Filoviridae family encompasses filamentous, non-segmented negative-sense RNA viruses, with natural reservoirs identified in various fish, reptiles, and mammals [155]. While humans are incidental hosts, zoonotic transmission of certain filoviruses can cause severe hemorrhagic fevers characterized by high mortality and significant epidemic potential [156]. The most prominent human pathogens within this family include Marburg virus (MBGV) and several species of Ebolavirus (EBOV), such as Zaire ebolavirus and Sudan ebolavirus [155].

A conserved feature among these viruses is the proteolytic cleavage of the envelope glycoprotein precursor (GP0) into the receptor-binding subunit GP1 and the fusion subunit GP2. This processing event is essential for the formation of the functional spike complex, which is responsible for host cell attachment and entry [156]. The presence of a canonical furin cleavage motif in GP0, highly conserved in pathogenic strains like MBGV and Zaire ebolavirus, initially suggested that furin-mediated processing was a critical determinant of virulence. This hypothesis was reinforced by the observation that Reston ebolavirus, which is non-pathogenic in humans, displays a less efficient furin cleavage site [157,158].

However, this model was challenged by reverse genetics studies. Mutant EBOV strains engineered to lack the furin cleavage site still accumulated the GP0 precursor but replicated to wild-type titers within days in cell culture [159]. More significantly, these furin-cleavage-deficient mutants retained full lethality in non-human primate models, demonstrating that furin processing is not essential for the profound pathogenicity of EBOV [160]. This apparent paradox (the conservation of a non-essential cleavage site) highlights a significant gap in our understanding and suggests that furin processing may confer a subtle, context-dependent advantage that has yet to be characterized. Potential non-essential roles for furin cleavage could include optimizing cell-to-cell spread in specific tissues, enhancing immune evasion by altering glycoprotein shedding or antigenicity, or promoting environmental stability of the virion.

A unique aspect of filovirus biology that may shed light on this puzzle is the expression of a soluble glycoprotein (sGP). This non-structural protein is the primary product of the GP gene and is secreted from infected cells in large quantities. Intriguingly, sGP is also a substrate for furin cleavage, yet the functional consequence of this event remains entirely unknown [161]. The processing of sGP by furin represents a compelling, non-canonical pathway that may influence pathogenesis through immunomodulation or by interfering with host immune responses. This presents a critical and under-explored area for future research into filovirus–host interactions.

### 5.7. Paramyxoviridae Family

The Paramyxoviridae family includes major human pathogens such as measles (MV), mumps (MuV), and respiratory syncytial virus (RSV), as well as highly virulent zoonotic agents such as the Nipah (NiV) and Hendra (HeV) viruses [162]. These viruses share a common architecture, characterized by a negative-sense, single-stranded RNA genome enclosed within a lipid envelope. This envelope is studded with two crucial glycoproteins: an attachment protein (variably designated G, H, or HN, depending on the virus) that binds to host cell receptors, and a fusion protein (F) that mediates the merger of the viral and cellular membranes, a process that can lead to the formation of characteristic syncytia [162,163].

A pivotal step in the paramyxovirus life cycle is the proteolytic activation of the F protein. It is translated as an inactive precursor, F0, which must be cleaved into the disulfide-linked subunits F1 and F2 to acquire fusogenic activity. For the majority of paramyxoviruses, including MV and MuV, this cleavage is efficiently catalyzed by the host protease furin within the trans-Golgi network (TGN) [164,165,166,167,168]. This intracellular activation equips nascent virions with a pre-triggered fusion machinery, allowing for direct, pH-independent viral entry at the plasma membrane and obviating the need for endocytosis [169]. This ‘ready-to-fuse’ state is thought to facilitate rapid and efficient cell-to-cell spread within the host, contributing to the high transmissibility characteristic of viruses like measles.

However, significant exceptions to this furin-dependent rule highlight evolutionary adaptations. The highly pathogenic Nipah and Hendra viruses possess F proteins with monobasic cleavage motifs that are not recognized by furin [170,171]. Instead, their activation is mediated by the endosomal/lysosomal protease cathepsin L, which requires viral internalization and endosomal acidification to trigger membrane fusion [170,172,173]. Respiratory syncytial virus (RSV) employs a hybrid strategy; its F protein undergoes an initial, priming cleavage by furin in the Golgi but achieves full fusogenic potential only after a second cleavage event post-secretion, which is mediated by extracellular or endosomal proteases [163,174]. This requirement for a second activation step mandates that RSV is internalized to complete its infectious entry [175].

The specific protease requirement for F protein activation is a key evolutionary adaptation that directly influences viral tropism, pathogenicity, and entry mechanisms across the Paramyxoviridae family. The delineation of these distinct pathways—from furin-dependent plasma membrane fusion to cathepsin L-dependent endosomal entry—provides a critical foundation for developing virus-specific therapeutic strategies, such as protease inhibitors tailored to block the essential activation step of a given pathogen.

### 5.8. Human Immunodeficiency Virus

The human immunodeficiency virus (HIV) is a retrovirus with a spherical structure that surrounds two identical, non-covalently linked, positive-sense single-stranded RNA strands. It is transmitted through bodily fluid and is characterized by progressive immunosuppression due to the depletion of CD4+ T lymphocytes, thereby increasing susceptibility to opportunistic infections. While HIV-1 exhibits global distribution and high pathogenicity, HIV-2, predominantly restricted to West Africa, causes less severe disease [176]. For practical purposes, references to HIV typically denote type 1 unless otherwise specified. The mature HIV virion is characterized by a conical core surrounded by a host-derived lipid envelope. Embedded within this envelope are the trimeric glycoprotein spikes, composed of the surface subunit gp120 and the transmembrane subunit gp41. These are produced by the proteolytic cleavage of a single precursor polypeptide, gp160, and form a non-covalent complex critical for receptor binding and membrane fusion [176].

The maturation of gp160 into its functional subunits (gp120-gp41) occurs predominantly in the TGN, where host proprotein convertases process it. Furin is the primary enzyme responsible for this cleavage, with proprotein convertase 7 (PC7) playing a secondary role [177,178,179]. The near-perfect evolutionary conservation of the arginine residues at the cleavage site (up to 99.7%) underscores the indispensable nature of this processing step for viral fitness [180]. This single proteolytic event arranges several critical outcomes for the virus: (1) it liberates the hydrophobic fusion peptide at the N-terminus of gp41 from the non-covalently associated gp120/gp41 heterodimers, which is essential for driving the merger of the viral and cellular membranes [181,182]; (2) it triggers a conformational change in the envelope trimer from an open, “loose” state to a stable, “closed” conformation that effectively shields key neutralization epitopes from the host’s immune system [180]; and (3) it ensures the proper glycosylation of gp120, reinforcing the glycan shield that protects the virus from antibody recognition. This cleavage is tightly coupled with the viral assembly pathway, as gp160 traffics through the secretory pathway to the site of virion budding, ensuring that newly formed particles incorporate the mature, fusogenic envelope spikes [183].

A paradoxical role of furin emerges in the context of HIV-associated neurocognitive disorders. Within the central nervous system, furin appears to exert a neuroprotective effect by processing and activating protease-activated receptors PAR1 and PAR2, which are upregulated in neuroinflammation [184]. This finding illustrates the complex duality of furin’s functions: it is an essential accomplice in viral propagation by activating the viral envelope protein, yet it can also modulate the host’s inflammatory response to mitigate neural damage. This nuanced understanding of furin’s dual roles—as both a viral facilitator and a host response modulator—is crucial for envisioning targeted therapeutic strategies that could inhibit its proviral activity without disrupting its potential beneficial functions in the host.

### 5.9. Flavivirus Genus

Flaviviruses, a genus within the Flaviviridae family, are a group of medically significant arthropod-borne viruses that pose a substantial global health burden. Prominent members include dengue (DENV), Zika (ZIKV), yellow fever (YFV), West Nile (WNV), and Japanese encephalitis (JEV) viruses [185]. These viruses share a common genomic organization, consisting of a single-stranded, positive-sense RNA that encodes three structural proteins—capsid (C), membrane (M), and envelope (E)—and seven non-structural (NS) proteins responsible for replication and immune evasion [186,187].

The assembly of infectious flavivirus particles is a carefully orchestrated process. Initially, the precursor membrane protein (prM) forms a heterodimer with the E protein, shielding it from premature fusion during transit through the acidic compartments of the secretory pathway. Virion maturation is finalized in the TGN through a critical two-step mechanism: first, the acidic pH of the TGN induces a major conformational rearrangement in the prM-E heterodimer, and second, this structural change exposes the prM cleavage site for processing by the host protease furin. This cleavage event severs the “protector” pr peptide from the mature M protein, resulting in the formation of infectious virions with fully functional E protein fusogens. Without furin cleavage, the prM peptide remains associated, sterically hindering the E protein from undergoing the conformational changes required for membrane fusion in the acidic environment of the endosome, thereby aborting the infection [188,189,190,191,192].

Although this cleavage can be incomplete—leading to the secretion of a fraction of immature particles, particularly in DENV infections—overwhelming evidence confirms that furin activity is indispensable for generating a fully infectious virion population [193,194,195]. The functional necessity of prM processing is unequivocally demonstrated by several lines of evidence: (1) specific furin inhibitors reduce infectivity by four orders of magnitude for WNV and over 1000-fold for DENV [196]; (2) engineered ZIKV with mutations in the cleavage site exhibit severely impaired replication in various cell cultures [197]; and (3) immature DENV particles produced in furin-deficient cell lines show reductions in infectivity-to-particle ratios of up to 10,000-fold [198]. A particularly compelling experiment demonstrated that the fusogenic activity of WNV, which is abolished in the absence of furin, can be fully rescued by the addition of exogenous enzymes, directly linking furin cleavage to membrane fusion competence [199].

The evolutionary conservation of this maturation step across the genus, combined with its profound impact on infectivity, positions furin as a prime candidate for the development of broad-spectrum antiviral strategies. Targeting this host-dependent mechanism could potentially yield therapeutics effective against multiple existing and emerging flaviviruses.

### 5.10. Coronaviruses

The Coronaviridae family encompasses a large number of species with diverse host ranges. Within this family, the Orthocoronavirinae subfamily includes seven viruses known to infect humans, the majority of which cause mild, common-cold-like illnesses. However, three zoonotic betacoronaviruses have emerged in the 21st century with significant epidemic and pandemic potential: Severe Acute Respiratory Syndrome coronavirus (SARS-CoV), Middle East Respiratory Syndrome coronavirus (MERS-CoV), and Severe Acute Respiratory Syndrome coronavirus 2 (SARS-CoV-2) [200,201]. These enveloped, positive-sense RNA viruses share a common structure defined by four main structural proteins: the nucleocapsid (N), membrane (M), envelope (E), and spike (S) protein. The trimeric S protein, which decorates the viral surface, is the primary determinant of infection, mediating host cell receptor binding, membrane fusion, and syncytia formation [200,202,203].

Proteolytic cleavage of the S protein is a conserved requirement for the infectivity of all three pathogenic coronaviruses. A key distinguishing feature of SARS-CoV-2 is the presence of a multibasic (RRAR) motif at the S1–S2 boundary, constituting a canonical furin cleavage site that is absent in SARS-CoV and other closely related sarbecoviruses [201,202,204]. Although the origin of this motif has been debated [205,206], furin-cleavable sites have a long evolutionary history in coronaviruses, with documentation in an avian coronavirus from 1954 and dozens of distinct sites identified in various animal coronaviruses prior to the COVID-19 pandemic. The critical consequence of this motif is that it enables proteolytic activation of the S protein during virus biogenesis in the producer cell, meaning SARS-CoV-2 virions are often released in a pre-activated state, unlike SARS-CoV, which relies more heavily on target-cell proteases for activation [207,208].

The S protein requires two cleavage events for full fusogenic activity: a first cleavage at the S1-S2 junction and a second cleavage at a site within S2 (S2′) that liberates the fusion peptide. For SARS-CoV-2 and MERS-CoV, a network of host proteases—including furin, TMPRSS2, and cathepsins—can mediate these cleavages [209,210]. Initial models posited that furin cleavage at S1-S2 was an absolute prerequisite for subsequent S2′ cleavage and entry. This was supported by data showing impaired S2′ processing and infectivity in cell lines with low cathepsin/TMPRSS2 activity when the furin site was mutated [210,211,212].

However, subsequent research revealed that in cells with high TMPRSS2 or cathepsin expression, the S protein can be activated efficiently through alternative pathways, rendering furin cleavage dispensable for infection in vitro [204,210,213,214]. Despite being non-essential, furin pre-cleavage at S1-S2 significantly enhances infectivity by priming the S protein, making it more susceptible to ACE2-induced conformational changes and subsequent S2′ cleavage [212,215,216]. The in vivo relevance of this priming is confirmed by studies in hamster and mouse models, where SARS-CoV-2 variants lacking the furin cleavage site cause attenuated disease compared to the wild-type virus [217].

While initially thought to be restricted to the S1-S2 junction, furin can also recognize and cleave S2′ when overexpressed at approximately 50-fold higher concentrations [218], a plausible in vivo scenario given the significantly elevated furin, presepsin, and IL-6 levels observed in COVID-19 patients [219]. This could create conditions favoring furin-mediated S2′ cleavage, potentially expanding SARS-CoV-2 tropism and contributing to disease severity.

Regulation of proteolytic processing through post-translational modifications adds another layer of complexity. In vitro studies reveal that O-glycosylation near the multibasic cleavage site inhibits furin cleavage, as illustrated by the Delta variant’s P681R mutation, which reduces glycosylation and enhances proteolytic processing [220,221,222]. Conversely, the Omicron variant (P681H) shows lower ACE2 affinity and reduced furin susceptibility [223,224,225], highlighting SARS-CoV-2’s evolutionary plasticity and the need for ongoing studies of virus–host interactions.

**Figure 7:**
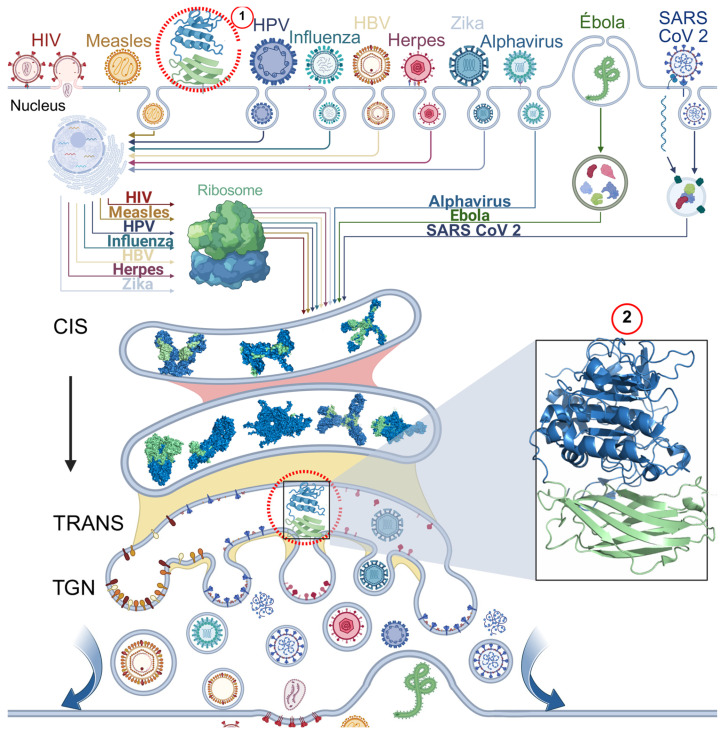
Schematic overview of viral entry and furin-mediated processing of viral proteins in the trans-Golgi network (TGN). The diagram depicts the cis- and trans-Golgi compartments, emphasizing the localization of furin within the TGN, where it cleaves viral precursor proteins at specific basic motifs. Representative viruses utilizing this pathway include: HIV (gp160 → gp120/gp41), measles virus (F0 → F1/F2), human papillomavirus (HPV L2 protein), influenza virus (hemagglutinin HA0 → HA1/HA2), and herpesviruses [glycoprotein B (gB) precursor]. These proteolytic events, occurring during transit through the TGN, are essential for viral maturation, membrane fusion capacity, and infectivity, Furin (PDB: 5JXG).

Given furin’s extensive involvement in viral replication, it is regarded as one of the most critical host proteins and a potential therapeutic target. The mechanisms of action of furin in different viruses are summarized below (Figure 7).

## 6. Genetic Variability of Furin and Its Implications in Viral Pathogenesis

Genomic studies have identified numerous variants in the FURIN gene that significantly influence viral infection processes, either through direct effects on viral protein processing or by altering key physiological pathways, including cardiovascular, metabolic, and immunological homeostasis [226,227]. The geographical distribution of these single-nucleotide polymorphisms (SNPs) reveals distinct population-specific patterns that may explain differences in viral infection susceptibility [228].

Recent comprehensive analyses have characterized approximately 7449 genetic variants across the FURIN locus, including 16 exonic and 4559 intronic variants. Among these, 635 represent missense mutations, 413 synonymous mutations, five deletions, two insertions, and 1825 variations in non-coding transcripts. Notably, 29 SNPs in the FURIN gene exhibit differential population distributions. The intronic SNP rs11073956 shows a significantly higher frequency in Southeast Asian populations than in other ethnic groups. Similarly, 17 SNPs exhibit marked frequency differences in African populations, comprising seven intronic SNPs, two in the 3′UTR region, two in the 5′UTR region, two in regulatory areas, and two intergenic SNPs [228].

**Figure 8:**
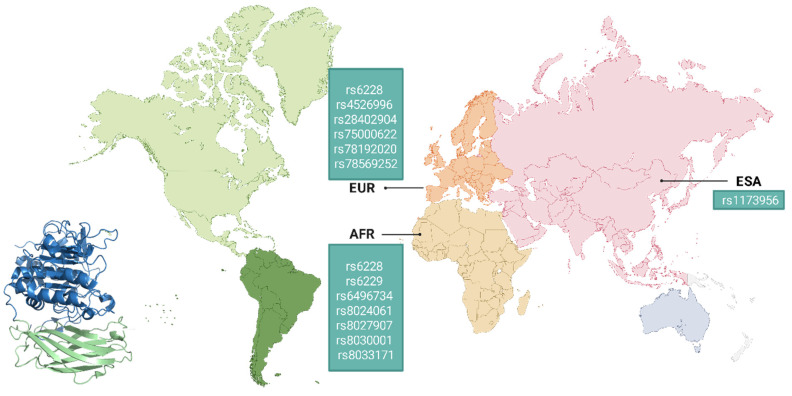
Known furin alleles in different regions of the world. The map highlights population-specific SNPs across different regions, including rs11073956, enriched in Southeast Asia; multiple intronic and regulatory variants in African populations; and characteristic intronic and 3′UTR variants in European populations. These differences may influence furin activity and contribute to variable susceptibility to viral infections. Figure Modified from Uddin MN et al. (2004), Furin (PDB: 5JXG) [227].

Comparative genomic analyses identified 10 SNPs with characteristic frequencies in European populations: three located in the 3′UTR region and seven in intronic areas [228]. These genetic variants may modulate furin expression and enzymatic activity, potentially contributing to interpopulation differences in susceptibility to metabolic disorders, which, in turn, may influence infectious disease outcomes. The heterogeneous distribution of these polymorphisms underscores the critical importance of considering population genetic context in association studies examining viral infection severity (Figure 8).

Proprotein convertases (PCs) play a pivotal role in regulating the infectivity and spread of enveloped viruses by proteolytically processing structural proteins, thereby exposing fusogenic domains essential for viral entry and replication. The emergence of novel cleavage sites in viral proteins has been consistently associated with enhanced pathogenicity, as demonstrated across multiple viral models. This phenomenon underscores the importance of molecular interactions between viral polybasic sequences and the host convertase system, where mutations in both the FURIN gene and viral recognition motifs can profoundly alter viral replication dynamics and infectious potential [229].

The COVID-19 pandemic has spurred unprecedented research into associations between furin genetic variants and viral susceptibility. Notably, the rs150925934 polymorphism enhances binding affinity between furin and the SARS-CoV-2 spike protein, thereby increasing proteolytic processing efficiency and elevating infection susceptibility. Equally significant is the rs6226 polymorphism, whose risk impact varies across populations, suggesting complex interactions with additional genetic and environmental factors modulating host response [228].

Computational predictions indicate that specific FURIN mutations (rs201172453 [R37C], rs148110342 [R81C], rs751909359 [R86Q], rs752639409 [R637Q], rs761541008 [R677W], rs35641241 [R745Q], rs193268286 [S685P]) may reduce enzymatic activity, potentially attenuating viral pathogenesis. These variants show higher prevalence in Qatar and Kuwait, countries that reported lower COVID-19 mortality rates, although socioeconomic factors and healthcare accessibility likely contributed to these outcomes [230].

Indian population studies revealed significant associations between the rs1981458 allele and COVID-19 mortality, with higher allele frequencies correlating with more severe outcomes. Functional analyses indicate that the rs1981458 variant primarily affects immune cells and antigen-presenting cells, potentially influencing viral processing and host immunity [231]. Similarly, the rs6226 variant (C/T + T/T genotypes) demonstrates consistent associations with COVID-19 susceptibility and disease severity through multiple linear regression analyses [228,230,232]. The G/C + C/C genotypes have shown significantly higher prevalence in COVID-19 patients compared to controls (OR: 2.750, 95% CI: 1.537–4.921) [232].

Experimental models using 293T-ΔFURIN knockout cell lines demonstrate that while furin is not required for SARS-CoV-2 spike protein processing, its absence in recipient cells substantially reduces cell–cell fusion kinetics. Interestingly, multinucleated cell formation persists, suggesting furin’s modulatory rather than deterministic role in syncytia formation and cytopathic effects [214]. Notably, several FURIN variants (rs753334944, rs16944971, rs73489557, rs6225) have shown no significant association with COVID-19 in Spanish populations [233].

In vitro studies reveal that plasmin-mediated cleavage of furin sites in the SARS-CoV-2 spike protein enhances viral infectivity by accelerating viral entry, membrane fusion, replication, and release processes. This finding carries clinical significance given elevated plasmin levels in COVID-19 patients with metabolic comorbidities [234].

Furin’s genetic influence extends beyond SARS-CoV-2. In Chinese HBV-endemic populations, the 229 C/T SNP in the FURIN P1 promoter region (1268 bp) has been associated with increased risk of chronic infection. The T allele increases transcriptional activity, elevating furin expression and subsequent HBeAg production, which decreases the likelihood of HBeAg seroconversion while thereby increasing the risk of chronic hepatitis and cirrhosis [235].

Regarding HPV susceptibility, genetic analyses of 875 women identified two protective FURIN SNPs: rs17514846 (*p* = 0.02622; OR:0.5896; 95% CI: 0.3725–0.9332) and rs2575712. The “AC” (rs17514846) and “AA” (rs2575712) genotypes conferred particular protection against HPV58 infection [236]. While mechanistic details remain unclear, these variants may alter furin’s proteolytic processing of the HPV L2 capsid protein, potentially interfering with viral replication. However, additional studies are needed to validate these findings and elucidate the underlying biological mechanisms (Table 1) [236].

## 7. Conclusions

In summary, furin plays a central role in the maturation and activation of numerous viral and host proteins, positioning it as a critical factor in both viral pathogenesis and cellular homeostasis. Its ability to cleave polybasic motifs in viral glycoproteins (such as the spike protein of SARS-CoV-2, the hemagglutinin of influenza, and the envelope glycoproteins of HIV and HPV) highlights its importance in facilitating viral entry, replication, and spread.

Despite significant advances, important questions remain, particularly regarding furin’s non-canonical roles in viral life cycles and its tissue-specific regulation. Future research should explore targeted inhibition strategies to disrupt furin-mediated viral activation without compromising its essential physiological functions. Additionally, investigating furin’s interactions with other host proteases and immune modulators could reveal novel antiviral interventions. Continued exploration of the molecular mechanisms of furin will be crucial in addressing current and emerging infectious diseases.

## Figures and Tables

**Table 1:** Summary of FURIN single-nucleotide polymorphisms (SNPs) associated with pathological processes.

Polymorphism	Protector/Pathogenic	Direct or Indirect	Virus or Comorbidity	Bibliographic Source
SNP229	Pathogenic	Direct	HBV	Lei et al., 2009 [235]
rs17514846	Protective	Direct	VPH	Zou et al., 2016 [236]
rs2575712	Protective	Direct	VPH	Zou et al., 2016 [236]
rs6226	Mixed	Direct	SARS-CoV-2	Uddin et al., 2024; Al-Mulla et al., 2021; (Elgedawy et al., 2024) [228,230,232]
rs150925934	Pathogenic	Direct	SARS-CoV-2	Uddin et al., 2024; Al-Mulla et al., 2021;(Elgedawy et al., 2024) [228,230,232]
rs201172453	Protective	Direct	SARS-CoV-2	Al-Mulla et al., 2021 [230]
rs8039305	Protective	Direct	SARS-CoV-2	Al-Mulla et al., 2021 [230]
rs1981458	Pathogenic	Direct	SARS-CoV-2	Pandey et al., 2024 [231]
rs6224	Pathogenic	Indirect	Hypercholesterolemia	Coto et al., 2022 [237]
rs4702	Pathogenic	Indirect	Hypercholesterolemia	Coto et al., 2022 [237]
rs17514846	Pathogenic	Indirect	Coronary heart disease	Zhao et al., 2018 [11]
rs753334944	N	N	N	Torre--Fuentes et al., 2021 [233]
rs16944971	N	N	N	Torre--Fuentes et al., 2021 [233]
rs73489557	N	N	N	Torre--Fuentes et al., 2021 [233]
rs6225	N	N	N	Torre--Fuentes et al., 2021 [233]
rs12917264	N	N	Hypotension	Cilhoroz et al., 2019 [238]
rs75493298	N	N	Hypotension	Cilhoroz et al., 2019 [238]
rs74037507	N	N	Increased systolic pressure	Cilhoroz et al., 2019 [238]
rs4702	N	N	Less cognitive impairment induced by radiotherapy	Yang et al., 2022 [16]

## Data Availability

No new data were created or analyzed in this study. Data sharing is not applicable to this article.
